# Metabolic syndrome before 16 weeks’ gestation: prevalence and haemodynamic profile in a socioeconomically disadvantaged pregnancy cohort: baseline findings from Mother’s Heart Study

**DOI:** 10.1007/s12020-026-04818-x

**Published:** 2026-10-17

**Authors:** A Mohebi, M Arstall, J Dalton, G Dekker, AR Alexander, MM Pathirana, M Wittwer, S Leemaqz, PH Andraweera

**Affiliations:** 1 School of Medicine, College of Health, Adelaide University, Adelaide, Australia; 2 Robinson Research Institute, Adelaide University, Adelaide, Australia; 3 Department of Cardiology, Lyell McEwin Hospital, Elizabeth Vale, Adelaide, Australia; 4 Department of Women’s Health, Lyell McEwin Hospital, Elizabeth Vale, Adelaide, Australia

**Keywords:** Metabolic syndrome, Cardio-metabolic risk factors, Pregnancy, Haemodynamic

## Abstract

**Background:**

Pregnancy provides an opportunity to identify young women at risk of future cardiometabolic diseases. We aimed to determine the prevalence of metabolic syndrome (MetS) and its components, and to assess haemodynamic profiles in early pregnancy.

**Method:**

We recruited pregnant women at Lyell McEwin Hospital before 16 weeks’ gestation. Haemodynamic parameters were measured using USCOM BP+ device. Blood glucose, lipid profile, liver and renal function were assessed. We included data from 264 women aged 19–44 years in the analyses. MetS was defined using the Harmonizing the MetS criteria.

**Results:**

The prevalence of MetS was 15.1% (*n* = 40). Individual MetS components included high waist circumference (97.3%, *n* = 257), high systolic blood pressure (4.5%, *n* = 12), high diastolic blood pressure (1.1%, *n* = 3), high triglycerides (47.7%, *n* = 126), low high-density lipoprotein cholesterol (15.2%, *n* = 40), and elevated fasting blood glucose (1.4%, *n* = 1). Compared with women without MetS, women with MetS had higher peripheral systolic blood pressure, central systolic blood pressure (cSBP), central diastolic blood pressure, central mean arterial pressure (cMAP), pulse rate (PR), and suprasystolic pulse rate, and lower suprasystolic pulse rate variability, pressure gradient, reflected wave transit time to the reflected wave peak, and pulse pressure. After adjustment for BMI and correction for multiple testing, cSBP, cMAP and PR remained independently associated with MetS (all *p* < 0.05).

**Conclusions:**

MetS in early pregnancy is associated with changes in cardiovascular haemodynamics, with adiposity contributing to these changes. Early screening for MetS during pregnancy may provide an opportunity to identify women at increased risk of future cardiometabolic disease.

**Supplementary Information:**

The online version contains supplementary material available at https://doi.org/10.1007/s12020-026-04818-x.

## Introduction

Cardiometabolic diseases, which include coronary artery disease and type 2 diabetes, are among the primary contributors to morbidity and mortality globally [1]. These diseases frequently share similar risk factors, including obesity, insulin resistance, hypertension, and dyslipidaemia. Identifying individuals with these risk factors is important to facilitate targeted early management to reduce morbidity and mortality due to cardiometabolic diseases [2]. Pregnancy provides an important opportunity to identify future cardiometabolic disease risk in young women.

Pregnancy has been described as a physiological “stress test” for the female cardiometabolic system, with substantial metabolic, inflammatory, and haemodynamic adaptations that may unmask underlying vulnerabilities [3]. Pregnant women with pre-existing cardiometabolic risk factors, including obesity, chronic hypertension, insulin resistance or dyslipidaemia, are at increased risk of experiencing further worsening of these conditions during pregnancy [4]. As a result, several international guidelines now recommend screening during and after pregnancy to identify women at increased risk of future cardiometabolic and cardiovascular disease.

Metabolic syndrome (MetS) is defined by a combination of cardiometabolic risk factors, which include central obesity, increased blood pressure, elevated fasting glucose levels, elevated triglyceride concentrations, and reduced levels of high-density lipoprotein cholesterol (HDL-C) [5]. Women with MetS are at increased risk of developing type 2 diabetes and cardiovascular diseases [6]. The prevalence of MetS and its components shows significant variation among different populations, influenced by genetic, lifestyle, and socioeconomic factors [7]. In the context of pregnancy, MetS has been associated with adverse pregnancy outcomes, including gestational diabetes mellitus (GDM), preeclampsia, fetal growth restriction, and preterm birth [8]. These complications not only impact immediate pregnancy but also carry long-term health consequences for the mother. For example, women experiencing these complications are at a higher risk of developing type 2 diabetes and cardiovascular diseases in the future [9].

Despite understanding the effects of poor metabolic health during pregnancy, there exists a lack of data that specifically addresses it in the early stages of pregnancy. Pregnancy is often the first point of engagement with the healthcare system for many young women, providing a unique opportunity for health assessment and intervention. This period offers an important opportunity for clinicians to deliver targeted health advice, identify modifiable risk factors, and initiate preventive strategies that may have long-term benefits for both maternal and child health. Women in early pregnancy are likely to comply with health recommendations [10] and routine contact with healthcare providers throughout pregnancy enables comprehensive counselling, screening, and early management of emerging issues. Therefore, identifying cardiometabolic risk factors in early pregnancy would provide a vital opportunity to reduce the long-term risk of cardiovascular diseases in young women. However, there is limited information on the prevalence of MetS and its associated cardiometabolic profile in early pregnancy. Previous studies have mainly focused on individual metabolic risk factors, with limited research on a combination of haemodynamic parameters, lipid profiles, liver and renal function in early pregnancy. As MetS affects multiple body systems, assessing these markers together may provide a more comprehensive understanding of the physiological changes associated with early pregnancy MetS. This may help identify early cardiometabolic changes that could support future screening and risk stratification of women at increased risk of cardiometabolic disease.

The primary objective of this study was to assess the prevalence of MetS and its individual components among pregnant women ≤ 16 weeks of gestation. The secondary objectives were (1) to compare haemodynamic profiles between women with and without MetS. (2) To compare liver and renal function between women with and without MetS.

## Materials and methods

The study design and methodology are described in the Mothers’ Heart Study protocol [11].

### Study design and setting

We conducted an observational prospective cohort study (Mother’s Heart Study) at the Lyell McEwin Hospital, Elizabeth Vale, in South Australia. This Northern Adelaide Local Health Network (NALHN) cohort includes women from a low socio-economic population with high rates of obesity, diabetes, and heart disease, which likely contribute to a high prevalence of MetS in early pregnancy. We recruited participants between December 2023 and December 2024, and were then followed up until six months postpartum. Before participation, pregnant women were provided with a flyer through the Pregnancy Online Platform (POPN) NALHN app, which contains detailed information regarding the study. The POPN app is specifically designed for pregnant women to register and submit their information before their initial antenatal appointment at the Lyell McEwin Hospital.

### Ethical considerations

Ethics approval was obtained from the Women’s and Children’s Health Network Human Research Ethics Committee (2023/HRE00084). All participants provided written informed consent before participating in the study. The study is registered with the Australian and New Zealand Clinical Trials Registry (ANZCTR) (Clinical trial number: ACTRN12625000756426; Registration date: 17 July 2025). Registration was completed retrospectively with the ANZCTR. Prospective registration was intended before recruitment commenced in December 2023; however, maintenance of the ANZCTR portal and associated technical difficulties prevented registration at that time. The study was registered at the earliest opportunity once these issues were resolved.

### Participants and recruitment

Pregnant women attending their first antenatal appointment ≤ 16 weeks of gestation were invited to participate in this study.

### Inclusion criteria

Any pregnant woman aged 18 years and older, attending their first antenatal appointment ≤ 16 weeks of gestation, was eligible.

### Data collection

We collected basic demographic information, including marital status, education, occupation, and household income. Socioeconomic status was assessed using the 2021 Index of Relative Socio-economic Disadvantage (IRSD) from the Australian Bureau of Statistics’ Socio-Economic Indexes for Areas (SEIFA), based on participants’ residential postcode. National IRSD decile rankings within Australia were used, where decile 1 represents the most socioeconomically disadvantaged areas in Australia and decile 10 represents the least disadvantaged areas. Medical history, family history, obstetric history, diet, exercise, smoking, and use of illicit substances were collected through a combination of patient self-reporting and medical record review. Psychological health was assessed because anxiety and depression have been associated with MetS and other cardiometabolic disorders. However, this relationship has been less well studied during pregnancy. During pregnancy, midwives routinely administer the Antenatal Risk Assessment Questionnaire (ANRQ). Scores range from 5 to 60. A total score of < 23 suggests a lower risk of anxiety; a score of ≥ 23 indicates a higher risk of anxiety. The Edinburgh Postnatal Depression Scale (EPDS) is used to assess the mental health of participants. Scores range from 0 to 30. A score of 0–9 suggests low depression, 10–12 indicates mild depression, and 13 or higher suggests significant depression. The results of these assessments were retrieved from the hospital medical records.

Height, weight, waist and arm circumference were measured. Body mass index (BMI) was calculated as weight (kg)/ height (m²). Obesity was defined as BMI ≥ 30 kg/m², and overweight as BMI ≥ 25 and < 30 kg/m² [9]. Waist circumference was assessed at the midpoint located between the lowest rib and the highest point of the iliac crest [12]. The arm circumference was measured at the midpoint of the upper arm, located between the shoulder and the elbow [13]. During pregnancy, the increasing size of the uterus limits the accuracy of waist circumference measurements after the first trimester. However, several studies have shown that measurements obtained during early pregnancy, particularly before 14–16 weeks of gestation, accurately reflect pre-pregnancy abdominal obesity and are therefore considered appropriate for research and clinical assessment of MetS in pregnancy [14].

### Haemodynamic parameters

Haemodynamic measurements were obtained non-invasively using the USCOM BP+, an automated oscillometric blood pressure device. Participants were seated and rested for 5 min before measurements were taken using an appropriately sized upper-arm cuff positioned at heart level. During cuff inflation above systolic pressure (suprasystolic pressure), the device recorded the brachial arterial pressure waveform. Pulse wave analysis was then used to estimate central blood pressure and other haemodynamic parameters. The haemodynamic variables measured using USCOM BP + are detailed in the Supplementary Appendix [15].

### Blood sample collection

Participants were given a pathology request form to attend a pathology collection centre for blood sample collection to assess the lipid profile (total cholesterol, triglycerides, and HDL), blood glucose, glycosylated haemoglobin (HbA1c), and liver and renal function tests at ≤ 16 weeks’ gestation. Although different pathology laboratories may use different analytical platforms for lipid measurements, laboratories in Australia are required to comply with national accreditation standards and participate in external quality assurance programs to ensure the accuracy and comparability of test results [16]. Non-fasting lipid measurements are considered reliable and have been shown to closely reflect fasting levels [17]. Therefore, non-fasting lipids were assessed. In early pregnancy, maternal lipid levels, such as cholesterol, triglycerides, and HDL, generally remain stable or show only minor decreases, with levels rising in the second trimester due to physiological changes [18]. Therefore, the lipid levels at or before 16 weeks are expected to reflect pre-pregnancy lipid levels [17].

### Diagnosis of metabolic syndrome

MetS was defined using Harmonizing the MetS criteria, which requires the presence of three or more of the following five components: (1) central obesity, characterised by elevated waist circumference, with different cutoffs depending on the population (≥ 80 cm for ethnicities including Asian, Europid, Caucasian, China, Middle Eastern, Central and South American and African), (≥ 88 cm for western guidelines including United States, Europe, and Canada) and (≥ 90 cm for Japanese women), (2) elevated triglycerides of ≥ 1.7 mmol/L, (3) reduced HDL cholesterol levels of < 1.3 mmol/L, (4) elevated blood pressure, systolic blood pressure (SBP) ≥ 130 mmHg or diastolic blood pressure (DBP) ≥ 85 mmHg, (5) fasting blood glucose level of ≥ 5.6 mmol/L [19]. For those who did not provide fasting samples, HbA1c was used, with a value of ≥ 5.7% to define elevated blood glucose [20].

### Statistical analysis

Data analyses were conducted using IBM SPSS version 29.0.1.0 (171), standard descriptive and inferential statistical methods. Data were reported as mean ± standard deviation (SD) for normally distributed continuous variables, median (interquartile range) for non-normally distributed variables, and frequencies (percentages) for categorical variables. The primary analysis involved estimating the prevalence of MetS ≤ 16 weeks’ gestation, defined using modified Harmonizing the MetS diagnostic criteria. For the secondary analysis, the prevalence of each MetS component, central obesity, elevated blood pressure, impaired blood glucose, high triglycerides, and low HDL cholesterol was calculated.

We compared haemodynamic, liver, and renal parameters between women with and without MetS using a non-parametric test (Mann–Whitney U tests) because the data were not normally distributed. Normality was assessed using the Shapiro-Wilk test and visual inspection of histograms. Categorical variables, including ethnicity, marital status, parity, employment status, smoking history, and previous medical or pregnancy complications among women with and without MetS, were compared using Chi-square (χ²) tests. Statistical significance was set at *p* < 0.05. To control for multiple testing, we used a false discovery rate (FDR) method with a 5% FDR (Q = 0.05). P-values were sorted in ascending order and matched with their respective Benjamini–Hochberg critical values. Variables with adjusted p-values that met the FDR criteria were deemed statistically significant [21]. We analysed haemodynamic variables, adjusted for BMI, using multiple linear regression.

## Results

A total of 325 participants were recruited to the Mother’s Heart Study. Of these, 264 completed the blood tests and were included in the analyses. Participants’ median age was 30 years (IQR 26–33) years. The median SEIFA score was 2 (IQR 1–2) and the median BMI was 27.57 kg/m^2^ (IQR 24.22–33.75). Most participants identified as Caucasian (67.4%), followed by Asian (21.6%), African (4.2%), and other ethnicities (6.8%). Of the participants, 56.8% were married, 13.3% were single, 28.4% were in de facto relationships, and 1.5% were divorced or separated. The majority of women (75%) were employed, and 69.3% had at least one previous child. The median gestational age at recruitment was 14.9 weeks (IQR 14.3–15.3). The median EPDS score was 6 (IQR 3–9), and median ANRQ score was 12 (IQR 8–21). Based on these screening scores, 7.6% of women were at high risk of depression, and 19.2% were at high risk of anxiety.

Previous major pregnancy complications, including hypertensive disorders of pregnancy, gestational diabetes mellitus, preterm birth and small for gestational age infants, were reported by 16.7%. Previously diagnosed medical conditions, including hypertension, diabetes, ischaemic heart disease, stroke or thromboembolism, were reported by 12.5% of the participants. A family history of type 2 diabetes, hypertension, ischaemic heart disease, stroke or thromboembolism was reported by 46.2%. Of the participants, 4.9% reported a history of smoking, and 80.8% reported engaging in physical exercise during pregnancy.

To assess potential selection bias, we compared baseline characteristics between women included in the analyses and those excluded because of missing blood test data (Supplementary Table 1). No significant differences were observed between included and excluded participants with respect to BMI, age, socioeconomic status (SEIFA), EPDS score, ANRQ score, ethnicity, marital status, employment status, previous pregnancy status, past medical history, family history of medical illness, smoking history and physical activity. Overall, the similarities across most baseline characteristics suggest that the analytical cohort was broadly representative of the recruited population.

### Prevalence of metabolic syndrome and its individual components among women ≤ 16 weeks of gestation

Of the 264 participants who completed all the assessments, MetS was diagnosed in 15.1% (*n* = 40) ***≤*** 16 weeks’ gestation. The most prevalent individual component was central obesity (97.3%), followed by elevated triglycerides (47.7%) and low HDL cholesterol levels (15.2%). Table 1 summarises the distribution of MetS and its individual components.

**Table 1:** Prevalence of metabolic syndrome and its individual components among women ≤ 16 weeks of gestation

MetS or its individual components	Yes (*n*%)	No (*n*%)
Metabolic Syndrome	40 (15.1%)	224 (84.8%)
Elevated Waist Circumference	257 (97.3%)	7 (2.6%)
Elevated Systolic Blood Pressure	12 (4.5%)	252 (95.4%)
Elevated Diastolic Blood Pressure	3 (1.1%)	261 (98.8%)
Elevated Triglycerides	126 (47.7%)	138 (52.2%)
Reduced HDL Cholesterol*	40 (15.2%)	223 (84.7%)
Elevated Fasting Blood Glucose**	1 (1.4%)	66 (98.5%)
Glycated haemoglobin A1c***	7 (6.7%)	97 (93.2%)

MetS components cutoffs: (1) Elevated waist circumference ≥80 cm, (2) elevated blood pressure SBP ≥130 mmHg or DBP ≥85 mmHg, (3) elevated triglycerides ≥1.7mmol/L, (4) reduced HDL cholesterol <1.3mmol/L, (5) elevated FBG ≥5.6mmol/L and HbA1c ≥5.7%

*HDL measurements were available for 263 participants

**FBG measurements were available for 67 participants

***HbA1c measurements were available for 104 participants

Results are in numbers and percentages

Due to the high prevalence of central obesity when using the ≥ 80 cm waist circumference threshold, a sensitivity analysis was performed using the ≥ 88 cm threshold recommended in western populations. Using this alternative cut-off, the prevalence of MetS decreased only marginally from 15.1% (40/264) to 13.6% (36/264). Waist circumference ranged from 73.0 to 160.0 cm, with a median of 99.5 (IQR 91.0–110.0) cm, demonstrating substantial variability within the cohort. Waist circumference and BMI were strongly correlated (Pearson’s *r* = 0.891, *p* < 0.001), suggesting these two measurements are closely linked indicators of adiposity, as detailed in Supplementary Table 1a.

Of the 264 participants, 67 (25.4%) provided fasting blood samples and 197 (74.6%) provided non-fasting blood samples. A sensitivity analysis restricted to participants with fasting blood samples demonstrated that 8 of 67 women met the criteria for MetS, corresponding to a prevalence of 11.9%, compared with 15.1% (40/264) in the overall cohort. The prevalence of elevated triglycerides among the fasting participants was 47.8% (32/67), compared to the overall cohort (47.7%; 126/264) (Supplementary Table 1b).

### Baseline characteristics of pregnant women with and without MetS

Baseline characteristics of pregnant women with and without MetS are presented in Table 2. Women with MetS were slightly older [median of 30.5 years (IQR 26.7–34 years)] compared to women without MetS [median of 29 years (IQR 26–33 years, *p* = 0.123)]. Women with MetS had a higher BMI, with a [median of 31.2 kg/m^2^ (IQR 26.53–37.37 kg/m^2^] compared to women without MetS [median of 27.19 kg/m^2^ (IQR 23.97–32.83 kg/m^2^, *p* = 0.004)]. Multiparity, history of smoking, previous major pregnancy complications, and previous medical illnesses were more common among women with MetS as compared to those women without MetS. However, the results were not statistically significant. No significant differences were observed between the two groups in socioeconomic status, ethnicity, marital status, employment, gestational age at recruitment, family history of medical conditions, ANRQ and EPDS scores.

**Table 2:** Baseline characteristics of pregnant women with and without MetS

Characteristic	Non-MetS [Median (IQR)] *N* = 224	MetS [(Median (IQR)] *N* = 40	*P* value
Age (median [IQR])	29 [26–33]	30.5 [26.7–34]	0.123
SEIFA score (median [IQR])	2 [1.0–2.0]	2.0 [1–2.0]	0.984
BMI (median [IQR])	27.19 [23.97–32.83]	31.2 [26.53–37.37]	0.004
EPDS (median [IQR])	5 [2–8]	5 [3–8.25]	0.685
ANRQ (median [IQR])	11 [8–20]	12.5 [8–20]	0.614
**Ethnicity**			
African	9 (4%)	2 (5%)	0.744
Asian	46 (20.5%)	11 (27.5%)	
Caucasian	153 (68.3%)	25 (62.5%)	
Others	16 (7.10%)	2 (5%)	
**Marital status**			
Single	29 (12.9%)	6 (15%)	0.93
Married	128 (57.1%)	22 (55%)	
Divorced	3 (1.3%)	1 (2.5%)	
De-facto	64 (28.6%)	11 (27.5%)	
**Employment**			
Employed	167 (74.6%)	31 (77.5%)	0.692
Not employed	57 (25.4%)	9 (22.5%)	
**Parity**			
0	73 (32.6%)	8 (20%)	0.112
≥ 1	151 (67.4%)	32 (80%)	
Gestational age (median [IQR])	14.3 [14.0–15.3]	15 [14.3–15.4]	0.996
Experienced previous major pregnancy complication/s*	34 (15.2%)	10 (25%)	0.125
No previous major pregnancy complication	190 (84.8%)	30 (75%)	
Presence of diagnosed medical condition**	26 (11.6%)	7 (17.5%)	0.299
No medical condition	198 (88.4%)	33 (82.5%)	
Positive family history of medical illnesses**	104 (46.4%)	18 (45%)	0.867
No family history of medical illnesses	120 (53.6%)	22 (55%)	
**History of smoking**			
Positive history of smoking	9 (4%)	4 (10%)	0.107
No smoking	215 (96%)	36 (90%)	
**Physical activity**			
Active exercise	181 (80.8%)	29 (72.5%)	0.23
No exercise	43 (19.2%)	11 (27.5%)	

EPDS, Edinburgh Postnatal Depression Scale

ANRQ, Antenatal Risk Questionnaire

*Gestational diabetes mellitus, gestational hypertensive disorders (preeclampsia, gestational hypertension), preterm birth, small for gestational age infants or intrauterine growth restriction

**Hypertension, type 2 diabetes mellitus, ischaemic heart disease, stroke, thromboembolism

Results are in median and interquartile range

### Haemodynamic profile of pregnant women with and without MetS ≤ 16 weeks of gestation

Haemodynamic parameters of women with and without MetS are detailed in Table 3. All results are reported as median and interquartile range. Compared to women without MetS, those with MetS had higher peripheral systolic blood pressure [111.5 mmHg (98.75–130.25) mmHg vs. 107 mmHg [98.25–116] mmHg, *p* = 0.048], central systolic blood pressure [103.5 mmHg (90.75–118.25) mmHg vs. 98 mmHg [87–106] mmHg, *p* = 0.012], central diastolic blood pressure [68.5 mmHg (62.25–75.25) mmHg vs. 64 mmHg [57–70] mmHg, *p* = 0.013], and central mean arterial pressure [86.5 mmHg (77–95.25) mmHg vs. 81.5 [73–88] mmHg, *p* = 0.006]. Pulse Rate (PR) was higher among women with MetS as compared to women without MetS [86 bpm (78.5–92.25) bpm vs. 77 (70–86), *p* = 0.002], Suprasystolic pulse rate (sPR) was higher among women with MetS compared to those without MetS [86.5 bpm (79.5–92.5) bpm vs. 79 bpm (72.2–87.75) bpm, *p* = 0.005]. Suprasystolic pulse rate variability (sPRV) was lower among women with MetS as compared to women without MetS [26.5 ms (18.75–52) ms vs. 37 ms (25–61.75) ms, *p* = 0.01]. Dp/Dtmax was lower among women with MetS compared to women without MetS [7.07 mmHg/s (5.39–9.45) mmHg/s vs. 8.99 mmHg/s (6.90–11.59) mmHg/s, *p* = 0.005]. sRWTTpeak was also lower among women with MetS compared to women without MetS [139 ms (127.25–148.5) ms vs. 145 ms (135–157.25) ms, *p* = 0.02]. Suprasystolic pulse pressure (sPP) was also lower in women with MetS compared to those without MetS [0.43 mmHg (0.32–0.54) mmHg vs. 0.52 mmHg (0.38–0.66) mmHg, *p* = 0.01]. Other parameters, including peripheral diastolic blood pressure, pulse pressure, mean arterial pressure, augmentation index, and sdP/dt artery, showed no significant difference between the two groups.

**Table 3:** Haemodynamic profile of pregnant women with and without MetS ≤ 16 weeks of gestation

Haemodynamic Parameter	Non-MetS [median (IQR)] *N* = 224	MetS [median (IQR)] *N* = 40	*P* value
pSBP	107 [98.25–116]	111.5 [98.75–130.25]	0.048
pDBP	65 [57–69]	66.5 [61–73]	0.067
cSBP	98 [87–106]	103.5 [90.75–118.25]	0.012
cDBP	64 [57–70]	68.5 [62.25–75.25]	0.013
PP	42 [37–48]	43 [39–57.25]	0.22
MAP	82 [75–88]	86 [76.25–95]	0.115
cMAP	81.5 [73–88]	86.5 [77–95.25]	0.006
PR	77 [70–86]	86 [78.5–92.25]	0.002
sPR	79 [72.2–87.75]	86.5 [79.5–92.5]	0.005
sPRV	37 [25–61.75]	26.5 [18.75–52]	0.01
AI	49 [38–63]	48 [35.75–69.77]	0.936
sdp/dt artery	736.32 [634.05–856.33]	778.89 [676.73–909.98]	0.324
Dp/Dtmax	8.99 [6.90–11.59]	7.07 [5.39–9.45]	0.005
sRWTTPeak	145 [135–157.25]	139 [127.25–148.5]	0.02
sSEP	288 [270–310]	276.5 [265 − 30]	0.053
sPP	0.52 [0.38–0.66]	0.43 [0.32–0.54]	0.01

Haemodynamic variables: pSBP, peripheral systolic blood pressure; pDBP, peripheral diastolic blood pressure; cSBP, central systolic blood pressure; cDBP, central diastolic blood pressure; PP, pulse pressure; MAP, mean arterial pressure; PR, pulse rate; sPR, suprasystolic pulse rate; sPRV, suprasystolic pulse rate variability; AI, augmentation index; sdp/dt artery, derivative of arterial pressure with respect to time; Dp/Dtmax, maximum suprasystolic pressure gradient; CMAP, central mean arterial pressure; sRWTTPeak, suprasystolic reflected wave transit time (peak); sSEP, suprasystolic systolic ejection period; sPP, suprasystolic pulse pressure

Results are presented as median and interquartile range

P-values are unadjusted. Benjamini–Hochberg false discovery rate correction was applied to account for multiple comparisons

### Associations between MetS and haemodynamic parameters after adjustment for BMI

Since obesity is known to influence haemodynamic changes and BMI differed significantly between women with and without MetS, multivariable linear regression analyses were undertaken to determine whether the observed haemodynamic differences remained associated with MetS after accounting for BMI. After adjustment, women with MetS had significantly higher central systolic blood pressure (cSBP; B = 6.04, 95% CI 0.76,11.32, *p* = 0.03), central mean arterial pressure (cMAP; B = 5.92, 95% CI 0.37,11.47, *p* = 0.04), and pulse rate (PR; B = 4.82, 95% CI 0.92,8.71, *p* = 0.02) compared with women without MetS. In contrast, the associations observed for the remaining haemodynamic parameters were no longer statistically significant after adjustment for BMI (Supplementary Table 2).

### Liver and renal function test results of pregnant women with and without MetS ≤ 16 weeks of gestation

Liver and renal function tests of women with and without MetS are presented in Table 4. Compared to women without MetS, those women with MetS had higher Gamma-Glutamyl Transferase (GGT) [median of 15 IU/L (IQR 11–22.25) IU/L vs. median of 12 IU/L (IQR 9–17) IU/L, *p* = 0.042]. The other liver and renal function results were not significantly different between the two groups.

**Table 4:** Liver and renal function tests of pregnant women with and without MetS ≤ 16 weeks of gestation

Laboratory Test	Non-MetS (Median [IQR]) *N* = 224	MetS (Median [IQR]) *N* = 40	*P* value
AST	19 [15.25–23]	18.5 [16–24]	0.937
ALT	15 [11–21.75]	15.5 [11.75–30.25]	0.688
GGT	12 [9–17]	15 [11–22.25]	0.042
LDH	174.5 [151.25–196]	167.5 [143.75–201.25]	0.416
AlkP	59 [46.25–74]	65.5 [59–76.5]	0.098
Total Bilirubin	5 [4–7]	5 [4–7]	0.74
Total Protein	68 [64.25–71]	68 [65–70]	0.632
Albumin	32 [29–34]	30 [28–32]	0.18
Globulin	36 [33–39]	38 [35–39.25]	0.585
BU	2.75 [2.2–3.2]	2.45 [2–3.3]	0.061
Creatinine	46.5 [41–51.75]	45 [40–50]	0.099
Sodium	137 [136–138]	136 [135–138]	0.055
Potassium	3.85 [3.7–4.1]	3.8 [3.7–4]	0.667

Liver and renal parameters: AST, aspartate aminotransferase; ALT, alanine aminotransferase; GGT, gamma-glutamyl transferase; LDH, lactate dehydrogenase; AlkP, alkaline phosphatase; BU, blood urea

Results are in median and Interquartile range

P-values are unadjusted. Benjamini–Hochberg false discovery rate correction was applied to account for multiple comparisons

To account for multiple comparisons, the Benjamini–Hochberg false discovery rate (FDR) correction was applied to the 29 haemodynamic, liver, and renal function parameters presented in Tables 3 and 4. Following FDR correction, the associations for PR, sPR, sPRV, Dp/Dtmax, cMAP, sPP, cSBP, and cDBP remained statistically significant, whereas GGT, pSBP and sRWTTpeak were no longer statistically significant after adjustment for multiple testing.

## Discussion

In our prospective cohort, MetS was prevalent among 15.1% of pregnant women at or before 16 weeks of gestation, indicating that a substantial proportion of women have cardio-metabolic risk factors in early pregnancy. Significant haemodynamic differences were observed in women with MetS compared to those without MetS.

Assessment of the individual components of MetS showed a high prevalence of central obesity and dyslipidaemia, with low rates of hypertension and elevated blood glucose. Central obesity was detected in 97.3% of the participants. Waist circumference measured at ≤ 16 weeks of pregnancy is a valid indicator of central obesity, as uterine growth does not significantly affect abdominal measurements until around 18 weeks of gestation [22]. These results are consistent with previous studies showing that central obesity is increasing among women of childbearing age, particularly among those living in lower socioeconomic areas. In Australia, people living in disadvantaged areas, such as the northern suburbs of Adelaide served by Lyell McEwin Hospital, tend to have higher rates of obesity than those living in more wealthy areas. This reflects ongoing differences in obesity prevalence and related health risks. Local data from the Northern Adelaide Local Health Network show a high prevalence of obesity (37%) and a greater burden of chronic disease risk factors, alongside social challenges [23, 24]. Abdominal adiposity plays an important role in metabolic dysfunction by contributing to insulin resistance, inflammation, vascular changes, and abnormal lipid levels. Our findings of a higher prevalence of elevated triglycerides and low HDL cholesterol are consistent with previous research from Europe, Asia, and North America, which has reported higher triglycerides and lower HDL cholesterol among pregnant women with central obesity [14, 25, 26]. These early lipid changes are associated with an increased risk of gestational diabetes, hypertensive disorders of pregnancy, large-for-gestational-age infants, and preterm birth. Pregnancy itself induces metabolic stress, while excess adiposity may further increase the risk of these complications [27, 28].

Although the prevalence of hypertension was low in this cohort, women with MetS showed significant haemodynamic differences compared with those without MetS. Women with MetS had higher pSBP, cSBP, and cMAP than women without MetS. After adjustment for BMI, cSBP and cMAP remained significantly associated with MetS. These differences in blood pressure may reflect increased central haemodynamic load and early alterations in vascular function [29]. Higher blood pressure in early pregnancy, even within the normal range, is associated with an increased risk of pregnancy-induced hypertensive disorders [30]. Although a 4 mmHg difference between 107 and 111 mmHg is unlikely to change clinical management for an individual woman, it may be clinically relevant at the population level [31]. Previous research in pregnancies affected by gestational diabetes and type 2 diabetes, gestational hypertension, and late-onset pre-eclampsia has linked these haemodynamic changes with impaired vascular function and arterial stiffness [32, 33]. Overall, these findings suggest that MetS in pregnancy may be associated with increased central haemodynamic load, with potential implications for pregnancy and postpartum cardiovascular health.

In addition, women with MetS showed significantly higher PR and sPR in the unadjusted analysis. However, the association with PR remained significant after adjustment for BMI, whereas the association with sPR was attenuated. These findings may reflect altered autonomic regulation and increased cardiovascular haemodynamic load [34]. This is consistent with a previous study by Hissen et al., which found that obese pregnant women had higher SBP and greater sympathetic nerve activity than normal-weight pregnant women, indicating increased cardiovascular load and sympathetic activation during pregnancy [35]. Similar haemodynamic changes have been reported in pregnancies affected by obesity and insulin resistance. These changes may contribute to increased cardiovascular demands during pregnancy and an increased risk of hypertensive disorders [36–39].

Women with MetS showed lower sRWTTpeak, sPRV, and sPP in the unadjusted analysis. These differences may indicate changes in arterial and haemodynamic function; however, they were no longer statistically significant after adjustment for BMI, suggesting that they may be substantially influenced by adiposity rather than independently associated with MetS. Higher levels of adiposity, particularly central adiposity, are associated with increased arterial stiffness and changes in vascular function related to insulin resistance, inflammation, and endothelial dysfunction [40]. Lower sRWTTpeak may indicate faster pulse wave transmission, which may reflect increased arterial stiffness and early vascular dysfunction [41]. This is consistent with previous research showing that women who later develop vascular complications may already have increased arterial stiffness in early pregnancy [42]. A prospective study found that women with higher carotid–femoral pulse wave velocity in the first trimester had a higher risk of developing pre-eclampsia, suggesting that arterial stiffness may be an early marker of pre-eclampsia [43]. Increased arterial stiffness in early pregnancy has also been associated with a greater risk of complications such as pre-eclampsia and may indicate early alterations in maternal vascular function [44]. Women with MetS also had lower Dp/Dtmax in the unadjusted analysis. This may reflect differences in cardiac function; however, the association was no longer statistically significant after adjustment for BMI. This suggests that adiposity may substantially influence the association between Dp/Dtmax and MetS.

MetS in pregnancy may also be associated with changes in liver function. Insulin resistance and chronic low-grade inflammation may contribute to liver fat accumulation and changes in liver function. Women with MetS had higher GGT levels in the unadjusted analysis, consistent with previous studies linking higher GGT levels to metabolic risk during pregnancy. Higher GGT levels have also been linked to an increased risk of hypertensive disorders and GDM. However, this association did not remain statistically significant after FDR correction. The association between GGT and metabolic complications may reflect common factors such as insulin resistance, inflammation, and changes in lipid metabolism. Despite this, the effect direction is consistent with previously reported metabolic, vascular, and hepatic alterations in pregnant women with metabolic risk, supporting the consistency of our findings with previous cohort studies. Similarly, sRWTTpeak and pSBP were no longer significant after FDR correction.

We acknowledge the following limitations. The study was conducted at a single hospital in South Australia, which may limit the generalisability of the findings. The sample size was relatively small, comprising 264 women. Lifestyle factors, including smoking, physical activity, and diet, were self-reported and may be subject to reporting bias. Measuring central obesity in early pregnancy also presents challenges. Although waist circumference is commonly used to estimate abdominal adiposity, variations in uterine size and body shape before 16 weeks of pregnancy may affect the accuracy of these measurements. Additionally, lipid levels, including triglycerides and cholesterol, naturally increase during pregnancy, and women with obesity may have elevated lipid levels even before conception. This makes it difficult to distinguish the effects of central obesity from normal pregnancy-related metabolic changes. Most participants provided non-fasting blood samples. Although the Harmonised criteria for MetS were originally derived using fasting samples, non-fasting lipid measurements are increasingly recognised as clinically acceptable and reflective of real-world practice, particularly for triglycerides, which show only modest postprandial variation in most individuals. To assess the potential impact of non-fasting sampling on our findings, we performed a sensitivity analysis restricted to participants who provided fasting samples. The prevalence of elevated triglycerides was similar in the fasting and overall cohorts (47.8% vs. 47.7%), while the prevalence of MetS was lower in the fasting subgroup (11.9% vs. 15.1%). These results suggest that fasting status did not substantially affect the prevalence of elevated triglycerides, although the small size of the fasting subgroup (*n* = 67) limits the precision of the sensitivity analysis. Our assessments were limited to early pregnancy (≤ 16 weeks), and postpartum follow-up will be necessary to determine whether women with MetS remain at increased cardiovascular risk. The Mothers’ Heart Study will include a 6-month postpartum follow-up, at which all MetS components will be assessed using fasting blood samples. This will allow for a more standardised evaluation of metabolic health outside the physiological context of pregnancy and allow direct comparison with established diagnostic thresholds.

The main strength of this study is its focus on women ≤ 16 weeks’ gestation, allowing metabolic risks to be identified early, before major physiological changes of pregnancy occur. Another strength is that all pregnant women were eligible for inclusion, making the findings more representative of women receiving care at tertiary hospitals serving socioeconomically disadvantaged populations. Measuring haemodynamic parameters early in pregnancy is also valuable, as it provides insight into cardiovascular changes at a relatively early stage.

## Conclusion

MetS in early pregnancy is associated with changes in cardiovascular haemodynamics, and adiposity may contribute to these changes. Early identification of MetS during pregnancy may help identify women at increased risk of future cardiometabolic disease and allow appropriate cardiovascular and metabolic follow-up.

## Supplementary Information

Below is the link to the electronic supplementary material.

Supplementary Material 1

Supplementary Material 2

Supplementary Material 3

Supplementary Material 4

Supplementary Material 5

## Data Availability

De-identified data can be provided upon reasonable request.
