# Supplementary material for: Metabolic syndrome before 16 weeks’ gestation: prevalence and haemodynamic profile in a socioeconomically disadvantaged pregnancy cohort: baseline findings from Mother’s Heart Study
Source: Endocrine. 2026 Oct 17;91(1):347. doi: 10.1007/s12020-026-04818-x (PMC13676646; doi:10.1007/s12020-026-04818-x)
Supplement: Supplementary file 1 [file 12020_2026_4818_MOESM1_ESM.docx]

# Supplementary Appendix

**Abbreviations, full names and definitions of USCOM BP+ variables.**

| Abbreviation | Full name | Definition |
| --- | --- | --- |
| CBP | Central blood pressure | Pressure in the central arteries (primarily the aorta) reflects the pressure experienced by the heart and central organs. |
| PBP | Peripheral blood pressure | Blood pressure is measured at the brachial artery. |
| MAP | Mean arterial pressure | The average arterial pressure during one cardiac cycle reflects organ perfusion. |
| PP | Pulse pressure | The difference between systolic and diastolic blood pressure reflects the force generated by each heartbeat and arterial elasticity. |
| PR | Pulse rate | The number of heartbeats per minute. |
| AI | Augmentation index | An index of arterial stiffness and wave reflection. |
| sPP | Suprasystolic pulse pressure | Derived from analysis of the suprasystolic brachial pressure waveform. |
| sPR | Suprasystolic pulse rate | Pulse rate derived from the suprasystolic pressure waveform. |
| sPRV | Suprasystolic pulse rate variability | Beat-to-beat variation in pulse rate derived from the suprasystolic pressure waveform. |
| sPPV | Suprasystolic pulse pressure variation | Beat-to-beat variation in pulse pressure derived from the suprasystolic pressure waveform. |
| sSEP | Systolic ejection period | The time the left ventricle ejects blood during each heartbeat. |
| sRWTTp | Suprasystolic reflected wave transit time to the reflected wave peak | The time required for the arterial pressure wave to travel to peripheral reflection sites and return to the aorta. |
| sdp/dt artery | Derivative of pressure with respect to time | The rate of change of arterial pressure over time reflects ventricular contractility and arterial properties. |
| Dp/Dtmax | Maximum suprasystolic pressure gradient | The maximum rate of pressure rise during systole reflects left ventricular contractility. |
