# Supplementary material for: Metabolic syndrome before 16 weeks’ gestation: prevalence and haemodynamic profile in a socioeconomically disadvantaged pregnancy cohort: baseline findings from Mother’s Heart Study
Source: Endocrine. 2026 Oct 17;91(1):347. doi: 10.1007/s12020-026-04818-x (PMC13676646; doi:10.1007/s12020-026-04818-x)
Supplement: Supplementary file 2 [file 12020_2026_4818_MOESM2_ESM.docx]

**Supplementary Table 1a: Sensitivity analysis of MetS prevalence using ≥88 waist circumference thresholds and distribution of waist circumference among pregnant women ≤ 16 weeks' gestation**

| **Variables** | **Value** |
| --- | --- |
| Participants with WC ≥80 cm, n (%) | 257 (97.3%) |
| Participants with WC ≥88 cm, n (%) | 221 (83.7%) |
| MetS prevalence using WC ≥80 cm, n (%) | 40 (15.1%) |
| MetS prevalence using WC ≥88 cm, n (%) | 36 (13.6%) |
| Waist circumference, mean (SD), cm | 102.0 (15.1) |
| Waist circumference, median (IQR), cm | 99.5 (91.0–110.0) |
| Waist circumference range, cm | 73.0–160.0 |
| Pearson correlation between WC and BMI | 0.891 |
| p-value | <0.001 |

**Supplementary Table 1b: Sensitivity analysis of MetS prevalence according to fasting status**

| **Variable** | **Overall cohort (n=264)** | **Fasting Subgroup (n=67)** |
| --- | --- | --- |
| Metabolic Syndrome | 40 (15.1%) | 8 (11.9%) |
| Elevated Triglycerides | 126 (47.7%) | 32 (47.8%) |
| High-density lipoprotein | 40 (15.2%) | 12 (17.9%) |
