# Supplementary material for: Metabolic syndrome before 16 weeks’ gestation: prevalence and haemodynamic profile in a socioeconomically disadvantaged pregnancy cohort: baseline findings from Mother’s Heart Study
Source: Endocrine. 2026 Oct 17;91(1):347. doi: 10.1007/s12020-026-04818-x (PMC13676646; doi:10.1007/s12020-026-04818-x)
Supplement: Supplementary file 4 [file 12020_2026_4818_MOESM4_ESM.docx]

# **Supplementary Table 1: Comparison of baseline characteristics between included and excluded participants.**

| **Characteristic** | **Included**  **[Median (IQR)]**  **N=264** | **Excluded**  **[(Median (IQR)]**  **N=61** | **P value** |
| --- | --- | --- | --- |
| Age (years), median (IQR) | 30 [26–33] | 28 [25–30.5] | 0.176 |
| SEIFA, median (IQR) | 2 [(1–2] | 1 [1–2] | 0.596 |
| BMI (kg/m²), median (IQR) | 27.57 [24.22–33.75] | 28.23 [24.17–32.00] | 0.649 |
| EPDS score, median (IQR) | 6 [3–9] | 6 [2.5–8] | 0.273 |
| ANRQ score, median (IQR) | 12 [8–21] | 11 [8–18] | 0.407 |
| Gestation (weeks), median (IQR) | 14.9 [14.3-15.3] | 14.9 [14.6-15.5] | 0.083 |
| **Ethnicity** | | | |
| African | 11 (4.2%) | 2 (3.3%) | 0.817 |
| Asian | 57 (21.6%) | 10 (16.4%) |  |
| Caucasian | 178 (67.4%) | 45 (73.8%) |  |
| Other | 18 (6.8%) | 4 (6.6%) |  |
| **Marital status** | | | |
| Married | 150 (56.8%) | 36 (59.0%) | 0.784 |
| De facto | 75 (28.4%) | 15 (24.6%) |  |
| Single | 35 (13.3%) | 8 (13.1%) |  |
| Divorced | 4 (1.5%) | 2 (3.3%) |  |
| **Employment** | | | |
| Employment (employed) | 198 (75.0%) | 44 (72.1%) | 0.643 |
| **Previous pregnancy** | | | |
| Previous pregnancy (yes) | 183 (69.3%) | 38 (62.3%) | 0.289 |
| **Previous pregnancy complication** | | | |
| Previous pregnancy complication* (yes) | 44 (16.7%) | 5 (8.2%) | 0.096 |
| **Previous Medical condition** | | | |
| Past medical history** (yes) | 33 (12.5%) | 6 (9.8%) | 0.564 |
| **Family history** | | | |
| Family history** (yes) | 122 (46.2%) | 27 (44.3%) | 0.783 |
| **Smoking status** | | | |
| Ever smoked (yes) | 13 (4.9%) | 3 (4.9%) | 0.1 |
| **Physical activity** | | | |
| Active exercise*** | 210 (80.8%) | 45 (17.3% | 0.436 |

EPDS**,** Edinburgh Postnatal Depression Scale

ANRQ**,** Antenatal Risk Questionnaire

***Pregnancy complications:** Gestational diabetes mellitus, gestational hypertensive disorders (preeclampsia, gestational hypertension), preterm birth, small for gestational age infants or intrauterine growth restriction

****Previous medical condition and family history:** Hypertension, type 2 diabetes mellitus, ischaemic heart disease, stroke, thromboembolism

***Active exercise data are available for 260 participants in the included group and 59 participants in the excluded group

Results are in percentages, medians, and interquartile ranges
