# Supplementary material for: Metabolic syndrome before 16 weeks’ gestation: prevalence and haemodynamic profile in a socioeconomically disadvantaged pregnancy cohort: baseline findings from Mother’s Heart Study
Source: Endocrine. 2026 Oct 17;91(1):347. doi: 10.1007/s12020-026-04818-x (PMC13676646; doi:10.1007/s12020-026-04818-x)
Supplement: Supplementary file 5 [file 12020_2026_4818_MOESM5_ESM.docx]

# Supplementary Table 2

Associations between MetS and haemodynamic parameters after adjustment for BMI

| **Haemodynamic parameter** | **BMI-adjusted B for MetS (95% CI)** | **P-value** |
| --- | --- | --- |
| pSBP | 3.56 (-0.48,7.59) | 0.08 |
| pDBP | 2.59 (-0.53,5.72) | 0.10 |
| cSBP | 6.04 (0.76,11.32) | 0.03 |
| cDBP | 5.65 (-0.47,11.76) | 0.07 |
| PP | 1.09 (-2.13,4.31) | 0.50 |
| MAP | 1.64 (-1.55,4.84) | 0.31 |
| cMAP | 5.92 (0.37,11.47) | 0.04 |
| PR | 4.82 (0.92,8.71) | 0.02 |
| sPR | 3.79 (-0.03,7.63) | 0.05 |
| sPRV | -13.82 (-36.46,8.82) | 0.23 |
| sAI | 0.77 (-7.90,9.43) | 0.86 |
| sDp/dt artery | 22.72 (-42.81,88.23) | 0.50 |
| sDpDtMax | -0.78 (-1.88,0.33) | 0.17 |
| sRWTTPeak | -4.83 (-11.99,2.35) | 0.19 |
| sSEP | -8.30 (-20.69,4.08) | 0.19 |
| sPP | -0.05 (-0.11,0.02) | 0.16 |

**Footnote:** Values are regression coefficients (B) from multivariable linear regression analyses adjusted for body mass index (BMI). B represents the adjusted mean difference in each haemodynamic parameter for women with MetS compared with women without MetS. Positive values indicate higher values, and negative values indicate lower values in the MetS group. CI, confidence interval.
